# The Role of Aerosol Liquid Water in Droplet-Assisted Ionization Mass Spectrometry

**DOI:** 10.1021/acs.analchem.5c04149

**Published:** 2025-08-31

**Authors:** Joshua Harrison, Kelvin M. Risby, Barnaby E. A. Miles, Thomas G. Hilditch, Jim S. Walker, Bryan R. Bzdek

**Affiliations:** School of Chemistry, 1980 University of Bristol, Cantock’s Close, Bristol BS8 1TS, United Kingdom

## Abstract

Chemical analysis of aerosols by mass spectrometry is challenging because aerosols contain little mass and have complex compositions. Consequently, relatively few approaches allow online molecular (i.e., minimal fragmentation) analysis on aerosols, particularly for ultrafine (<100 nm) particles. Droplet-assisted ionization (DAI) mass spectrometry is a promising and straightforward approach for aerosol molecular analysis. In DAI, liquid aerosol droplets are delivered directly to the mass spectrometer inlet, where the droplets break up and form molecular ions similar to those observed in electrospray ionization. This study explores how aerosol liquid water, serving as a matrix, controls ion generation in DAI for systems spanning simple binary and more complex ternary ones. The results demonstrate that ion yields are tightly coupled to the aerosol’s hygroscopic response, leading to humidity- and phase-dependent ionization efficiencies. When the water to analyte molar ratio is less than ∼2, ion formation is relatively insensitive to water content, but once the matrix to analyte molar ratio exceeds ∼2, ion yield is highly sensitive to aerosol liquid water. These dependencies can be easily minimized by normalizing the environmental relative humidity experienced by the aerosol immediately before delivery to the MS inlet. This study enables a critical evaluation of the factors controlling ionization by this inlet-based approach and identifies experimental designs that will facilitate a more widespread implementation of DAI for aerosol chemical analysis in different application domains. Lastly, this work is an interesting example of how knowledge of aerosol physicochemical properties can answer questions about ionization mechanisms in mass spectrometry.

## Graphical abstract

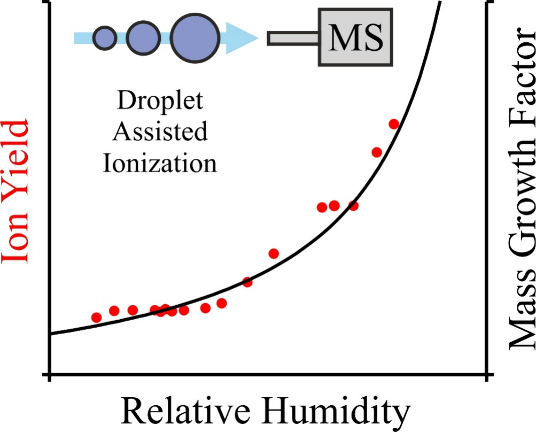

Aerosol particles are ubiquitous, representing important contributors to air pollution and the largest uncertainty in predictions of future climate.^1^ They may also serve as the vehicles for disease transmission.^2^ Additionally, aerosol-based approaches for efficient synthesis of chemicals and nanomaterials are increasingly common.^3–6^ Appropriate chemical analysis instrumentation is central to evaluating the unique roles aerosols play in all of these important applications. While much instrumentation has been developed to characterize the chemical composition of aerosols by mass spectrometry (MS),^7–10^ comparatively few online approaches provide direct information about aerosol molecular composition.^11–14^ Moreover, molecular analysis of ultrafine (<100 nm) particles remains relatively uncommon.^15,16^

Inlet ionization approaches are a recently discovered class of ionization method where the molecules within a sample can be ionized and chemically analyzed without the need for a dedicated ion source, lasers, or high voltage.^17–22^ Inlet ionization approaches inherently rely on aerosols to generate gas-phase ions from condensed-phase molecules.^23–25^ For example, in solvent-assisted ionization (SAI), a solution containing analyte is aspirated directly into the MS inlet through a fused-silica capillary, effectively generating analyte-containing liquid droplets.^18^ Within the inlet, these droplets break up, generating charge that ultimately migrates to the analyte. In matrix-assisted ionization (MAI), solid particles containing matrix and analyte are introduced into the MS inlet using several different methods, with analyte ions formed through sublimation, shattering, or triboelectric processes involving these particles.^17,26^

Because these approaches are easy to implement and ions arise directly from aerosols, inlet ionization approaches are now increasingly used for aerosol molecular analysis. MAI has been adapted to analyze the composition of the surface layers of submicrometer organic aerosol by condensation onto a solid core containing an organic acid.^27–29^ This approach provides insight into the surface composition of atmospherically relevant particles and low volatility condensable species. Droplet-assisted ionization (DAI), where liquid aerosol droplets are sampled directly into the MS inlet through a capillary, has been used to study submicrometer plumes of aerosol,^30–33^ as well as levitated picoliter droplets.^34^ The approach has provided insights into the size-dependent chemical composition of secondary organic aerosol generated under atmospherically relevant conditions, with smaller particles exhibiting compositions consistent with nucleation from highly oxidized molecules and larger particles providing evidence for particle-phase processing.^35^ DAI has also been used to explore enhanced reaction kinetics in aerosol droplets relative to bulk solution.^33^

However, widespread usage of these approaches for aerosol chemical analysis requires a more detailed understanding of the dependencies of ion formation on analyte composition and environmental conditions. Ions generated by DAI are similar to those observed by ESI of similar systems,^30^ and ionization efficiencies are likely molecule-specific.^36^ More importantly, in DAI the droplet solvent (typically water) is thought to be the source of ions. In nearly any application where aerosol composition measurement is desirable, relative humidity (RH) has the potential to substantially modulate the aerosol liquid water content,^37^ potentially impacting the absolute and relative abundances of the detected ions. Aerosol hygroscopic response can be highly nonlinear, particularly at high RH values.^38^

This work explores how ion formation in DAI is affected by environmental RH for binary (solute–water) and ternary (two solutes–water) droplets. Separately, the aerosol hygroscopic response is both measured with an electrodynamic balance and predicted using thermodynamic models. Integrating measurements of RH-dependent ion formation from aerosol droplets with estimations of the aerosol’s hygroscopic response provides clear evidence for how water serves as a matrix that facilitates the formation of analyte ions from liquid droplets. These RH-derived matrix effects can be easily avoided by briefly exposing the aerosol plume to a supersaturated RH environment immediately before analysis, facilitating practical usage of the approach.

## Experimental Section

The role of aerosol hygroscopic response on ion formation and ion identity during DAI was experimentally investigated.

### DAI-MS Experiments

The experimental setup is illustrated in Figure 1. In the first stage of the experimental setup (Figure 1a), polydisperse aerosol was generated from aqueous solutions using an atomizer (TSI 3073) at a 4 L min^–1^ flow rate. Investigated binary (solute–water) systems included angiotensin II (>99%, ApexBio), hydrocortisone (≥98%, Sigma–Aldrich), ammonium sulfate (≥99.0%, Sigma–Aldrich), cytochrome C from equine heart (≥95%, Sigma–Aldrich), and equine heart myoglobin (≥90%, Sigma–Aldrich). Additionally, an equimolar mixture of ammonium sulfate and angiotensin II was studied. The solutions were made using HPLC plus grade water (Sigma–Aldrich). Angiotensin II, hydrocortisone, and ammonium sulfate were selected as model systems, because they exhibit very different hygroscopic responses. The two proteins were selected because the range of charge states they exhibit upon ionization can depend on the ionization process.

**Figure 1:**
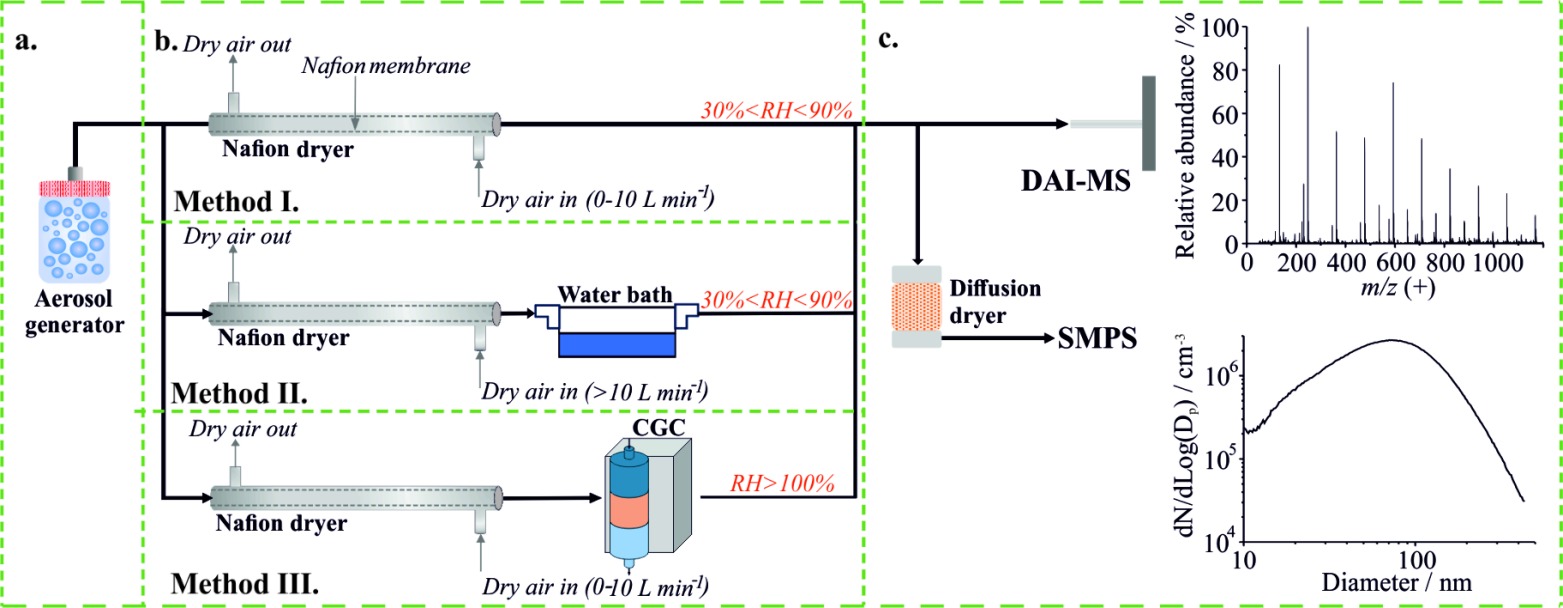
Experimental setup describing (a) generation of submicrometer aerosol through atomizing a solution, (b) RH manipulation of the aerosol flow, and (c) chemical and size distribution analysis of the RH-conditioned aerosol.

In the second stage of the experimental setup (Figure 1b), the atomized wet aerosol was conditioned to a desired RH through one of three different methods. The goal was to test how changes in RH conditioning and conditioning history (i.e., droplet water content and phase) alter the sensitivity of the DAI analysis. In Method I, the wet aerosol was dried to a desired RH (30%–90%) by passing it through a Nafion dryer (Model MD-700–24S-3, Perma Pure) with a user-selected sheath gas flow rate (0–10 L min^–1^). In Method II, the initially wet aerosol was first dried to <30% RH within the Nafion dryer and then rehumidified by flowing the dried aerosol over deionized water held within a temperature-controlled water bath. The final RH was controlled by altering the water bath temperature, with higher temperatures generating higher RHs. In Method III, the wet aerosol was dried to a desired RH in a similar manner to that described in Method I, but the RH-conditioned aerosol was then sent to a condensational growth chamber (CGC, Series 110A Spot Sampler, Aerosol Devices). The CGC grows aerosol particles by water condensation in a supersaturated environment to liquid droplets 1–3 μm in diameter.^39^ Considering that the dry size of the aerosol is typically 25–300 nm, particle growth in the CGC, in principle, normalizes the amount of water within a droplet before chemical analysis. The RH of the aerosol flow was measured with a probe (PTU300, Vaisala, ±1% accuracy for RH values of 0–90%, ± 1.7% for RH values of 90%–100%). The aerosol residence time within the RH conditioning setup was relatively long (>1 s), compared to the aerosol’s equilibration time to a new RH (approximately tens of milliseconds), so we expect that the aerosol will have fully equilibrated to the desired RH upon exiting the conditioning setup.

In the third stage of the setup (Figure 1c), the RH-conditioned aerosol flow was split. One portion of the flow was sent directly to a quadrupole time-of-flight MS (Waters Synapt XS) via a custom inlet made of a stainless-steel capillary (54 mm length, 1 mm OD, 0.5 mm ID). The source block temperature was kept at 70 °C. The flow rate into the MS was ∼2 L min^–1^. Rapid thermal or aerodynamic breakup of the liquid aerosol droplets upon delivery to the MS inlet leads to the production of molecular ions, which are mass analyzed by time-of-flight (TOF). Mass spectra were collected in sensitivity mode and, upon analysis, represented in centroid mode. Another portion of the flow was dried with a diffusion dryer and then sampled by a scanning mobility particle sizer (SMPS), which consists of an electrostatic classifier (Model 3082, TSI), differential mobility analyzer (Model 3081, TSI), and condensation particle counter (Model 3789, TSI). The SMPS flow rate is 1.5 L min^–1^. The SMPS measurement permits quantification of the number and mass concentration of the dried aerosol. For the aerosol generated in this study, dry particle size distributions were polydisperse with modal number diameters typically around 45 nm. The remaining aerosol flow was HEPA-filtered and vented into a fume cupboard.

The ion yield was defined as the number of analyte ions detected per analyte molecule delivered to the MS inlet and was calculated using eq 1:

1 $$\mathrm{Ion yield}=\frac{M_{R}\times \mathrm{IC}}{Q_{M}\times N_{A}}$$

where *M*_R_ is the analyte molar mass (μg mol^–1^), IC is ion count (counts min^–1^), *N*_A_ is Avogadro’s constant (molecule mol^–1^), and *Q*_M_ is the analyte flow rate into the MS (μg min^–1^). The IC parameter was quantified by summing all selected ions across 60 × 1 s acquisitions. For the small molecules, clusters, and peptides, monoisotopic masses associated with the analyte and with ion intensity >5% relative abundance were included in the IC. *Q*_*M*_ was quantified from the SMPS-measured aerosol mass concentration of dry solute (*M*, μg m^–3^) and MS flow rate (*Q*_g_, m^3^ min^–1^) using eq 2:

2 $$Q_{M}=M\times Q_{g}$$

Uncertainties in ion yield are dominated by variability in ion signal intensity and so were calculated by dividing the time-dependent signal intensity for each ion of interest during a 2 min experiment into 24 5-s windows and then taking the standard error across those 24 windows.

### Aerosol Hygroscopic Response

Aerosol hygroscopic response is represented by the mass growth factor (MGF):

3 $$\mathrm{MGF}\left(\mathrm{RH}\right)=\frac{M_{\mathrm{wet}}\left(\mathrm{RH}\right)}{M_{\mathrm{dry}}}$$

where *M*_wet_(RH) is the mass of the particle at a specific RH and *M*_dry_ is the mass of the particle at 0% RH. In this way, MGF provides a quantitative measure of the amount of aerosol liquid water in a particle. For the binary angiotensin II system and the ternary ammonium sulfate-angiotensin II system, MGF was first measured experimentally using a comparative kinetics–electrodynamic balance (CK-EDB).^40^ The CK-EDB method is conceptually illustrated in Figure S1. Individual charged droplets with initial radius of ∼30 μm are generated with a microdroplet dispenser (Microfab, MJ-ABP-01, 30-μm orifice diameter) and then trapped between two cylindrical electrodes in a RH-controlled chamber. The trapped droplet is illuminated by a 532-nm laser (Laser Quantum, Ventus CW), while a CCD camera collects elastically scattered light across scattering angles of 35°–60° (phase function, Figure S1b). The phase function is then used to retrieve the droplet radius with ∼200 nm accuracy. The response of droplet radius to controlled changes in RH is quantified and converted to MGF by accounting for droplet density (Figure S1c). Droplet density as a function of RH was estimated using the Aerosol Inorganic–Organic Mixtures Functional groups Activity Coefficients (AIOMFAC) model.^41^ Measurements of the hygroscopic response of hydrocortisone were also attempted, but this system is poorly soluble and quickly crystallized during CK-EDB experiments, even at high RH, suggesting that hydrocortisone’s hygroscopicity is minimal.

Additionally, aerosol hygroscopic response for all systems was estimated using thermodynamic models. MGF for ammonium sulfate was calculated using the Extended Aerosol Inorganics Model (E-AIM),^42^ whereas MGF for angiotensin II, hydrocortisone, and the ternary mixture was predicted using AIOMFAC.^41^ The MGF thermodynamic predictions compared very favorably against the CK-EDB measurements (see Figure S2) and confirmed experimental inferences about the poor hygroscopic response of hydrocortisone. Therefore, in the discussion below, the experimental ion yields are compared against predicted MGF values.

## Results and Discussion

DAI is an inlet ionization approach that enables molecular analysis of aqueous aerosol droplets with minimal net charge by sampling them directly into the capillary inlet of a MS. Gas-phase molecular ions are thought to arise by either aerodynamic or thermal breakup of the droplets within the inlet capillary, which has a temperature and pressure differential.^30,31^

### DAI Mass Spectra

Figure S3 provides example DAI mass spectra for three systems investigated in this study (angiotensin II, hydrocortisone, and ammonium sulfate aerosol at 80% RH). In general, DAI mass spectra exhibit singly or multiply charged molecular ions with little fragmentation (similar to mass spectra generated using electrospray ionization). For angiotensin II (Figure S3a), the primary detected ions are the singly charged and doubly charged molecular ions at 1046.5 *m*/*z* and 524.1 *m*/*z*, respectively. For hydrocortisone (Figure S3b), the main detected ions correspond to the singly charged molecular ion (363.5 *m*/*z*) and singly charged hydrocortisone clusters at 726.0 *m*/*z* and 1088.4 *m*/*z*. Ammonium sulfate aerosol droplets (Figure S3c) generate ammonium bisulfate clusters spanning a wide range of *m*/*z* values.^43^ To estimate the ion yield for this system, all singly and doubly charged ammonium bisulfate clusters <1200 *m*/*z* (i.e., those labeled in Figure S3c with a relative ion intensity >5%) were included in the calculation. Because this system generates clusters across a very wide range in *m*/*z*, necessarily the ion yields are an underestimate of the true value. However, most detected ion intensities were in the <1200 *m*/*z* range examined.

### RH-Dependent Ion Yields for Binary Systems

Previous studies have found that a protic solvent (e.g., water, polypropylene glycol) is essential to forming molecular ions in DAI.^30,31^ However, these studies only explored conditioning the aerosol plume under wet and dry conditions or replacing water with an aprotic solvent (e.g., acetonitrile). Figure 2 shows the measured ion yield and corresponding MGF for angiotensin II (Figure 2a), hydrocortisone (Figure 2b), and ammonium sulfate (Figure 2c) aerosol across an RH range spanning 30%–95%. Importantly, ion yields for each analyte were measured after RH conditioning the aerosol plume using two different methods. In one set of experiments (red symbols in Figure 2), the aerosol flow was partially dehumidified by passing it through a Nafion dryer (Method I in Figure 1b). In a second set of experiments (blue symbols in Figure 2), the aerosol was first fully dehumidified in a Nafion dryer before being rehumidified to a desired RH by passing it over a temperature-controlled water boat (Method II in Figure 1b).

**Figure 2:**
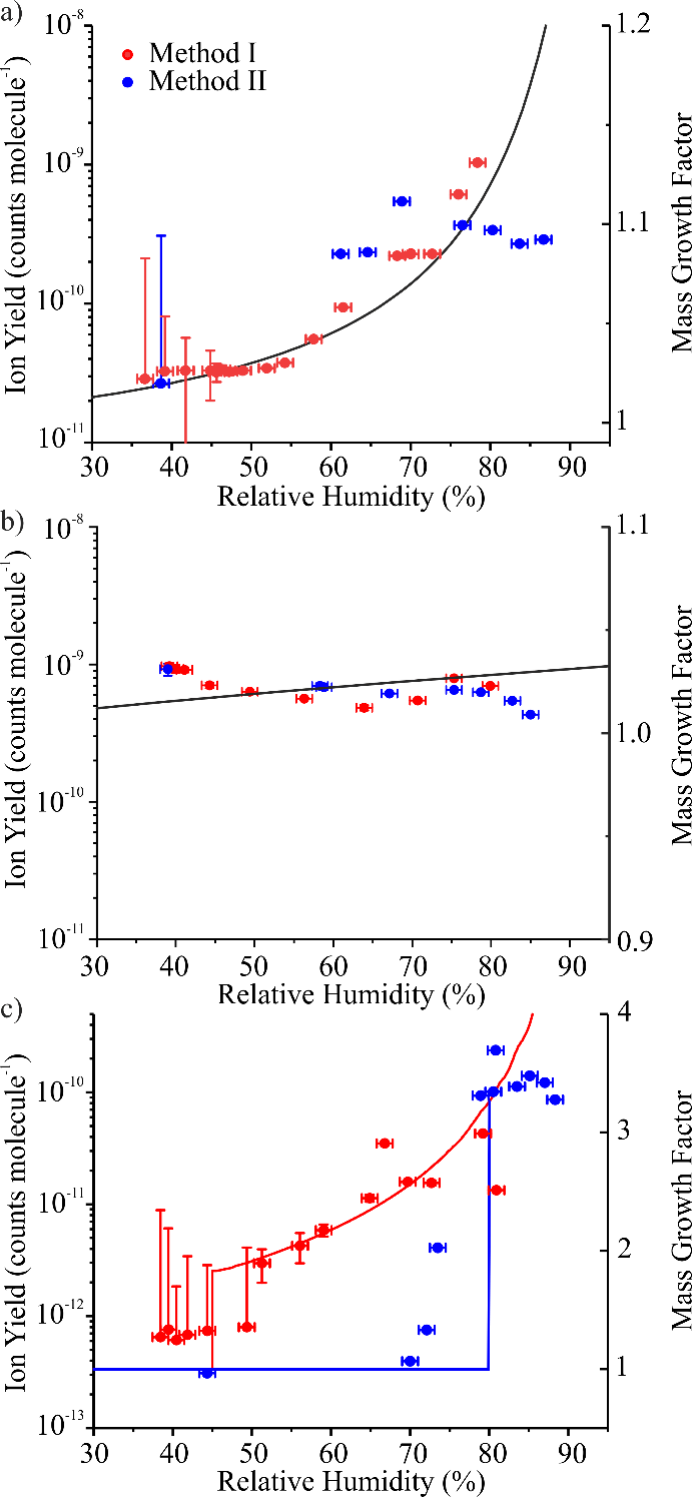
DAI-MS measured ion yields using Method I (red symbols) and Method II (blue symbols) to condition the RH, along with the corresponding MGF (lines) for a) angiotensin II, b) hydrocortisone, and c) ammonium sulfate. For angiotensin II and hydrocortisone, MGF was calculated using AIOMFAC. For ammonium sulfate, MGF was calculated using E-AIM.

Figure 2 broadly shows that ion yield can vary 2–3 orders of magnitude, depending on the RH at which the aerosol is conditioned. However, these variations are clearly linked to the chemical system being investigated. For angiotensin II, the ion yield increases with increasing RH, with a particularly steep increase in the observed ion yield when RH is above ∼65%. Moreover, the ion yield is independent of the RH conditioning history (i.e., using Method I or Method II). In contrast, hydrocortisone exhibits virtually no RH dependence to ion yield, although, similar to angiotensin II, the ion yields are independent of the RH conditioning history. Finally, ammonium sulfate exhibits a complex RH dependence to its ion yield. Using Method II, where droplets are first dried to <30% RH and then rehumidified before introduction to the MS inlet, ion yield is low and roughly invariant up to an RH of ∼70%. Then, the ion yield steeply increases over a very short RH range before exhibiting further, more modest, increases in ion yield when RH is greater than ∼80%. In contrast, when Method I is used to condition the aerosol (i.e., wet droplets are either directly sampled by the MS or are partially dried by passing them through the Nafion dryer), a very different trend is observed. Beginning at high RH, where ion yields are roughly consistent with those measured using Method II, the ion yield decreases with decreasing RH. However, a steep decrease in ion yield is observed around 50% RH. At RH values <50%, ion yield is low and invariant with further decreases in RH and is of a similar order of magnitude to (although slightly higher than) the values measured using Method II.

The different RH dependencies to ion yields for these different systems can be reconciled with an understanding of their hygroscopic response, which is quantified through the MGF (solid lines in Figure 2). MGF provides the relative amounts of solute and solvent in a particle at a given RH. When MGF≫1, a substantial amount of water is contained within the particle. When MGF = 1, the particle is completely dry.

For angiotensin II (Figure 2a), MGF exhibits a smooth change with RH, with a shallower change in MGF at lower RH values and a much steeper change as RH increases above ∼65%. These observations are broadly consistent with the experimental measurements of ion yield. An increase in MGF from 1.05 (∼60% RH) to 1.10 (∼75%) corresponds to a 5× increase in ion yield, highlighting the sensitivity of ion yield to MGF. Hydrocortisone (Figure 2b) is nearly insoluble in water (solubility limit around 310 mg L^–1^ at 37 °C), meaning its MGF only minimally increases from 1 at higher RH values, consistent with the lack of RH-dependence to ion yield. For an anhygroscopic compound such as this, the water content does not appreciably change as the aerosol is conditioned to different RH values, leading to an RH-independent ion yield. Lastly, ammonium sulfate (Figure 2c) exhibits a hysteresis in phase, depending on its RH conditioning history. For an initially dry particle, MGF = 1 until the deliquescence relative humidity (∼80% RH) is reached, where the particle undergoes a phase change to a liquid droplet.^44^ As RH increases further, droplet size also increases. These trends in ammonium sulfate MGF are consistent with the trends in the ion yield experiments conducted using Method II, though the observed deliquescence RH is a bit smaller, potentially due to small amounts of impurity in the sample.^45^ For an initially wet droplet, MGF decreases smoothly with decreasing RH until the efflorescence RH is reached. At the efflorescence RH, the droplet undergoes a phase change from a liquid droplet to a solid particle. Below the efflorescence RH, MGF = 1. Literature values for ammonium sulfate efflorescence RH cover a relatively broad range (e.g., ∼31%–48% RH).^44^ The efflorescence behavior is consistent with the ammonium sulfate experiments conducted using Method I, although the measured efflorescence RH (∼50% RH) is slightly higher than expected, possibly due to minor sample impurities.^45^ Comparing across all experiments, these results suggest that DAI-MS ion yields are tightly coupled to each system’s respective hygroscopic response.

Figure 2 demonstrates that the protic solvent water acts as a matrix that facilitates charge transfer to analyte molecules within the droplet. To better quantify the role of water, Figure 3 plots ion yield against *n*_water_/*n*_analyte_ (calculated from MGF), where *n*_water_ is the number of moles of water in the droplet and *n*_analyte_ is the number of moles of analyte, for the three systems shown in Figure 2, focusing on RH-conditioning Method I. For angiotensin II, ion yield is roughly invariant with *n*_water_/*n*_analyte_ at values less than ∼2. Given the lack of any discontinuity in the MGF plot (Figure 2a), at these low values for water content, the aerosol is likely to be supersaturated with respect to solute but still liquid, rather than crystalline. When *n*_water_/*n*_analyte_ > 2, a power-law relationship between ion yield and water content is observed. A similar trend is observed for ammonium sulfate, except that the overall ion yields are lower and the power-law relationship starts at a higher *n*_water_/*n*_analyte_ value, due to the phase change caused by the efflorescence process at lower RH, which leads to the creation of a solid particle. (The data point with ion yield ∼10^–12^ and *n*_water_/*n*_analyte_ ≈ 6.6 corresponds to the data point at ∼50% RH in Figure 2c, where the aerosol has clearly undergone efflorescence before the MGF curve predicts it should.) For hydrocortisone, which is nearly anhygroscopic, all data points are clustered around *n*_water_/*n*_analyte_ values between 0.1 and 1, with little sensitivity to ion yield with changing *n*_water_/*n*_analyte_. Note that even though hydrocortisone is nearly anhygroscopic, water is still essential for ionization, as changing the atomizing solvent to a nonprotic one like acetone reduced the ion yield by 2–3 orders of magnitude relative to the protic solvent water.

**Figure 3:**
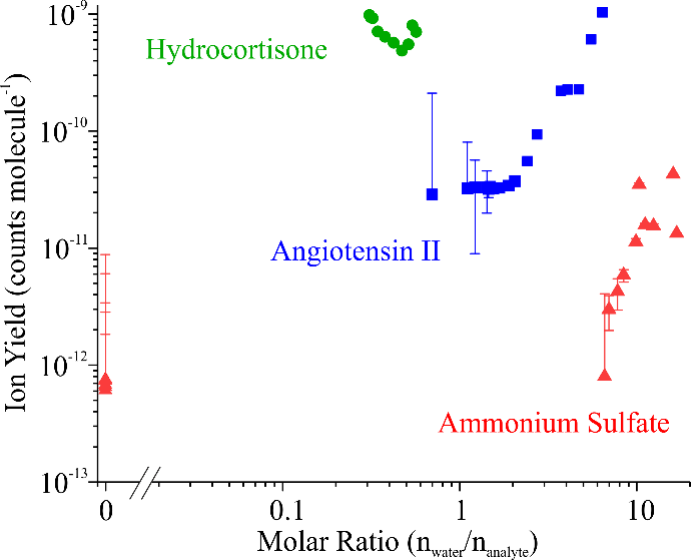
Ion yield plotted against *n*_water_/*n*_analyte_ for angiotensin II (blue), hydrocortisone (green), and ammonium sulfate (red). For all three systems, *n*_water_/*n*_analyte_ was varied using Method I (i.e., a plume of liquid aerosol droplets was dried to a desired RH).

### RH-Dependent Ion Yields for Multicomponent Aerosol

So far we have examined simple binary (solute-water) systems. To evaluate whether aerosol hygroscopic response continues to influence ionization efficiency in more complex, multicomponent systems, RH-dependent ion yields for an equimolar mixture of ammonium sulfate and angiotensin II were quantified (Figure 4a), using Method I for RH conditioning. The individual ion yields for ammonium sulfate and angiotensin II, as well as the total ion yield, are shown. There are several conclusions to draw from this figure. First, the ion yields increase continuously as RH increases. Additionally, ion yields for ammonium sulfate and angiotensin II are largely similar (i.e., within a factor of ∼2) for the same RH, with ammonium sulfate exhibiting marginally but consistently higher ion yields than angiotensin II. Comparing to the MGF data (solid line), the trend in ion yield is generally consistent with trends in the aerosol hygroscopic response. The presence of angiotensin II suppresses the expected efflorescence-induced phase change in ammonium sulfate at low RH. Consequently, ionization at low RH is more efficient than for a solid particle due to a greater solvent content within the aerosol.

**Figure 4:**
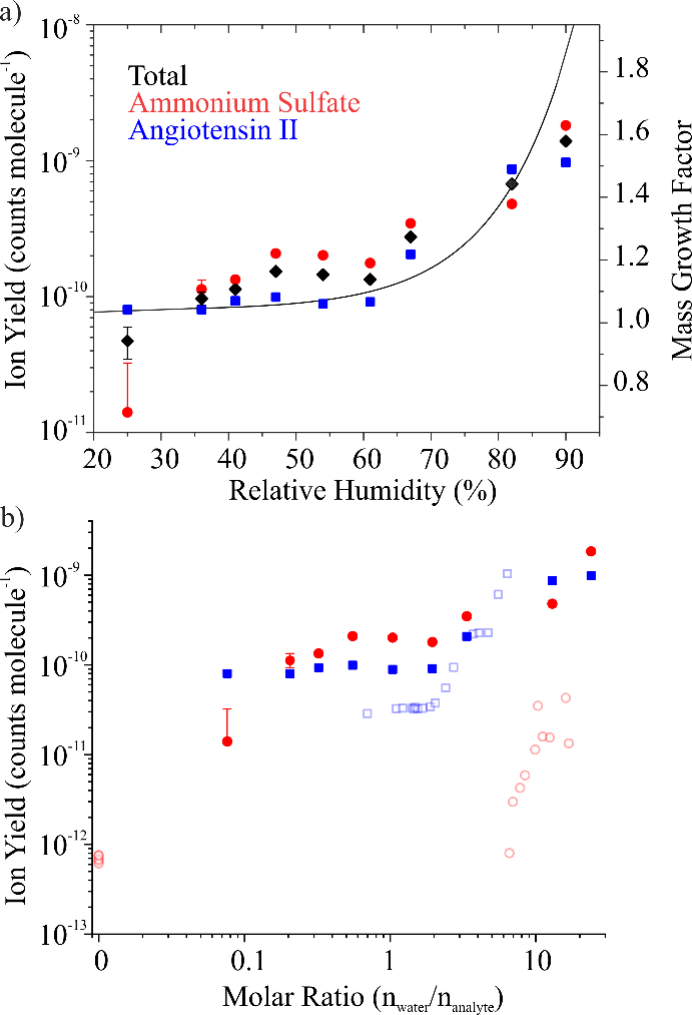
(a) Total ion yield and ion yields for both components of the equimolar ammonium sulfate-angiotensin II mixture aerosol as a function of RH. MGF (solid line) is plotted against the right *y*-axis. (b) Ion yields as a function of *n*_water_/*n*_analyte_ for the same system. In panel (b), data for the corresponding binary systems (angiotensin II and ammonium sulfate) are also shown for comparison (open symbols).

These observations are further confirmed by plotting *n*_water_/*n*_analyte_ for the mixture system and comparing to the binary systems (Figure 4b). At *n*_water_/*n*_analyte_ < 2, ion yields are roughly invariant with droplet water content but are generally higher than those observed for angiotensin II or ammonium sulfate on their own. An explanation for this observation (relative to ammonium sulfate, which would be crystalline at these RH values) is that the aerosol plume consists of liquid droplets containing some water (i.e., higher MGF relative to binary ammonium sulfate). However, the ion yield for angiotensin II in the mixture at *n*_water_/*n*_analyte_ < 2 is also higher than that for the binary angiotensin II system, suggesting the presence of salt ions may also facilitate more efficient ionization. (This observation is consistent with a MAI study finding that addition of ammonium salts increased the analyte ion abundance.^46^) At *n*_water_/*n*_analyte_ > 2, the ion yield increases with increasing water content, although the gradient of change is not as large as observed for the binary systems.

### Effect of RH on Ion Charge State

The above has demonstrated that a protic solvent can exhibit a strong influence on analyte sensitivity using DAI. We now show that the protic solvent also affects the charge state of the detected ions. For these experiments, angiotensin II and the proteins cytochrome C and myoglobin were atomized in binary solutions, RH conditioned using Method I, and analyzed by DAI-MS. Angiotensin II typically presents in the mass spectrum as either [M + H]^+^ or [M + 2H]^2+^ (see Figure S3a). In contrast, the proteins can exhibit a wide range of charge states (Figures S4 and S5). Figure 5 shows that the extent of multiple charging of the analyte increases with increasing RH. Although the change is fairly modest for angiotensin II, given only two charge states are typically observed, the difference is more significant for the two proteins. Between 53% and 90% RH, the weighted average charge state increases from 2.9 to 4.4 and 4.0 to 6.7 for cytochrome C and myoglobin, respectively. This observation is consistent with the expected role of the protic solvent in the ionization process. At higher RH, the droplet water content is significantly larger. Consequently, more elemental charges are generated upon ionization and a greater fraction of the analytes receive multiple charges. At lower RH, the droplet water content is reduced, so the droplets are less efficient at generating charge upon breakup. In addition to fewer molecules receiving any charge, the overall charge imparted to the analyte is reduced. An interesting observation for myoglobin is that at 90% RH, the heme-bound (holo-) form of the protein is more abundant in the mass spectrum (84% relative abundance, calculated from the sum of signals from holomyoglobin divided by the sum of signals from both holo- and apo-myoglobin) than at 53% RH (56% relative abundance, see Figure S4), suggesting DAI may preserve more native-like protein structures.

**Figure 5:**
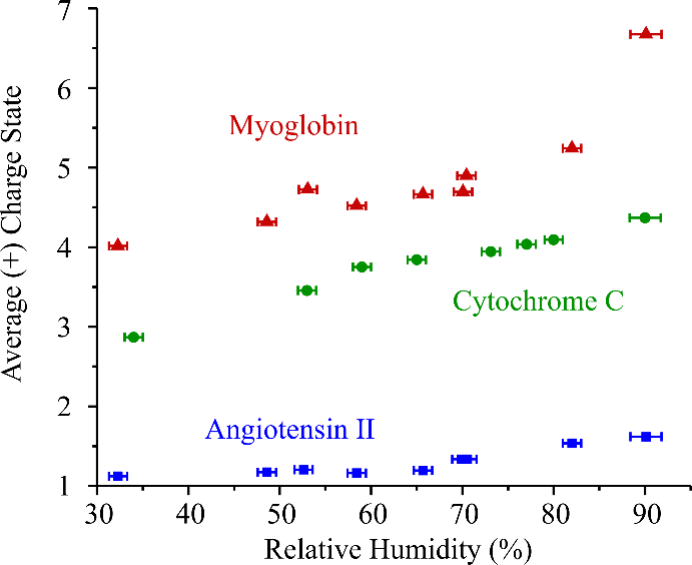
Average charge state observed for angiotensin II, cytochrome C, and myoglobin in DAI mass spectra, as a function of RH.

### Overcoming RH-Dependent Sensitivity by Condensational Growth

DAI-MS is a convenient approach for molecular analysis of aerosols in a range of contexts. The above discussion highlights how the RH conditioning history of the aerosol can influence the sensitivity of the analysis. This sensitivity to water content could be nonideal for applications where RH is not held constant (e.g., ambient measurements or flow tube experiments aimed at exploring RH-dependent chemistry). However, these sensitivity challenges may be overcome by conditioning the aerosol directly before introduction to the MS using a condensational growth chamber (CGC, Method III in Figure 1b). The CGC grows particles as small as 5 nm to droplets 1–3 μm diameter by passing them through a supersaturated RH region.^39^ In Figure 6, ion yields where the aerosol is RH-conditioned using Method III are shown for all systems studied in Figures 2 and 4, alongside measurements performed using RH-conditioning Method I and (for ammonium sulfate only) Method II. The first conclusion is that exposing the aerosol to a very high RH immediately prior to MS analysis removes any dependence of ion yield on the original RH conditioning, further supporting the conclusion that water is essential to the ionization process. Moreover, for the hygroscopic compounds, the ion yields are significantly higher when passed through the CGC, even when initially conditioned at high RH. This observation is because supersaturated RH values are reached in the CGC, ultimately leading to higher water content in the droplet. While the RH of the aerosol plume immediately before being sampled into the MS is not directly measurable and is unlikely to be supersaturated, it likely is >95%, which is higher than that accessible in the RH conditioning measurements previously described. Given the steepness observed in aerosol hygroscopic response at high RH, a relatively small increase in RH can yield a significant increase in sensitivity. The only system for which ion yield does not increase substantially when the CGC is placed in line is hydrocortisone, which is expected, given its anhygroscopic nature.

**Figure 6:**
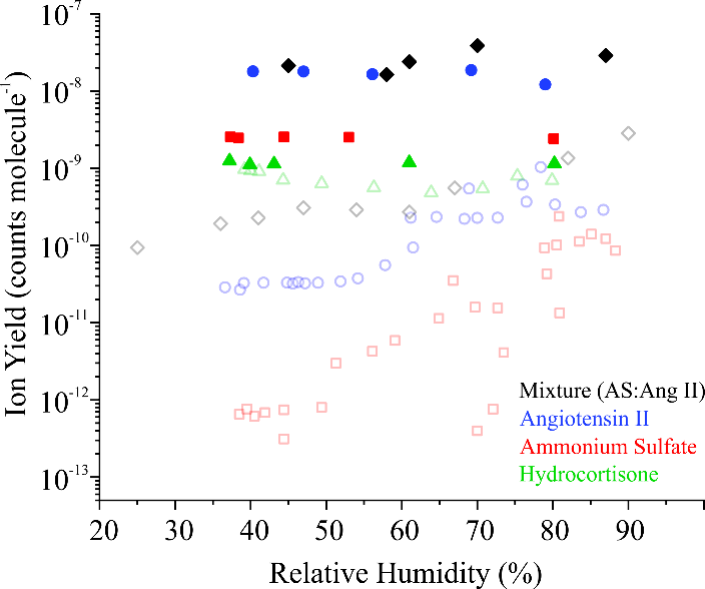
Comparison between ion yields for RH-conditioned particles after supersaturation via CGC (filled symbols) and without (open symbols) for angiotensin II, hydrocortisone, ammonium sulfate, and the equimolar mixture of ammonium sulfate and angiotensin II. Error bars are smaller than the symbols.

### Mechanistic Insights Into the Ionization Process

The results of this study, combined with previous DAI studies, facilitate development of a relatively complete picture of the mechanism of ion generation in DAI. Capillary temperature, droplet size, and solvent (i.e., matrix) properties are the three parameters that most significantly influence ion yields.

#### Capillary Temperature

The highest ion yields are typically observed at inlet temperatures >500 °C, whereas the lowest ion yields are observed at 150–300 °C (with relatively higher ion yields at inlet temperatures <150 °C).^31^ The lower ion yields at these midrange temperatures are thought to arise from slower solvent evaporation than at higher temperatures. The relatively higher ion yields at temperatures <150 °C may arise due to droplets freezing and shattering in the gas expansion while traversing the capillary inlet. Computational fluid dynamic modeling coupled with a droplet evaporation model enabled mechanistic insights into the droplet evaporation process within the heated capillary inlet and found that at inlet temperatures <150 °C, droplets largely undergo aerodynamic breakup, but the probability of aerodynamic vs thermal droplet breakup is highly dependent on temperature.^32^ However, at inlet temperatures >150 °C, thermal droplet breakup dominates and the observed temperature dependence to ion signal intensity can be explained in relation to ion formation processes (i.e., charge residue or ion evaporation models). While capillary temperature can be varied through custom-designed inlets (thereby increasing ion yields by up to ∼1–2 orders of magnitude), implementing the highest capillary temperatures (>500 °C) can be challenging in practice due to safety considerations.

#### Droplet Size

Smaller droplets are more efficient vehicles for ionizing analytes than larger droplets. Kerecman et al. found that the number of ions generated from a wet droplet exiting a CGC is relatively constant, regardless of the dry particle size that was sent into the CGC, suggesting that the number of gas phase ions depends on the size of the droplet introduced to the MS.^35^ This observation is consistent with droplet size-dependent ion yields that were measured as part of this study. In Figure S6, aerosol containing angiotensin II was equilibrated to 70% RH (by Method I in Figure 1b), size selected either by an aerodynamic aerosol classifier (Cambustion, Ltd.) or a differential mobility analyzer, and then sampled directly into the MS. Therefore, in this experiment, *n*_water_/*n*_analyte_ was constant across all measured particle sizes. Figure S6 shows that smaller droplets generated higher ion yields (by nearly 2 orders of magnitude) than larger droplets. Moreover, in DAI-MS studies of picoliter volume droplets (5–50 μm radius) where all droplets contained the same analyte concentration (i.e., *n*_water_/*n*_analyte_ is constant), smaller droplets also more efficiently ionized the analyte than larger droplets (by ∼1 order of magnitude).^34^ Lastly, in an approach where solid carboxylic acid particles (∼100–300 nm diameter) enabled surface-selective chemical composition measurements, smaller particles enabled more efficient ionization than larger particles due to ion signal intensity scaling with particle surface area.^27^ These trends are broadly consistent with ESI studies finding generation of smaller droplets by electrospray results in more-efficient ionization.^47,48^

#### Solvent Properties

Previous DAI-MS studies demonstrated that the presence of a protic solvent (e.g., water, polypropylene glycol) led to significantly higher ion yields compared to using an aprotic solvent (e.g., acetonitrile).^30,31^ Moreover, higher water content was generally correlated with better signal, although those experiments only explored wet droplets, dried particles, and droplets generated from a 50/50 mixture of water and acetonitrile.^31^ This study demonstrates that ion yields are tightly linked to the hygroscopic response of the sample being analyzed, with ion yields spanning 3 orders of magnitude, depending on the aerosol liquid water content. Only a few water molecules per analyte molecule are required to dramatically increase ion yield. This observation also holds for more complex samples containing multiple analytes, although the magnitude of the increase in ion yield with increasing *n*_water_/*n*_analyte_ is smaller, likely due to an averaging out of properties in the mixture. Our results are also consistent with previous observations that smaller (dry) particles give higher ion yields than larger (dry) particles.^35^ In that experiment, size-selected dry particles were grown in a CGC to a uniform wet droplet size before sampling into the MS. The droplet size dependence to ion yield can therefore be interpreted in the context of *n*_water_/*n*_analyte_ values: upon introduction to the MS, the droplets grown from the smallest dry particles would have higher *n*_water_/*n*_analyte_ ratios than the droplets grown from the largest dry particles. Moreover, in a separate study where picoliter volume droplets were chemically analyzed using DAI-MS,^34^ ion yields were also strongly dependent on *n*_water_/*n*_analyte_: for droplets of the same size, lower solute concentrations (i.e., larger *n*_water_/*n*_analyte_ values) generated higher ion yields. To contrast the role of water (where only a few molecules are required per analyte molecule to obtain good signal), consider the commonly used inlet ionization matrix 3-nitrobenzonitrile.^49^ In previous DAI-MS experiments where aqueous droplets were doped with 3-nitrobenzonitrile, a matrix-to-analyte ratio of 1000:1 (i.e., a large excess of 3-nitrobenzonitrile) in liquid droplets with moderate water content was required to match the ion yield for the same sample without matrix but at a higher water content.^31^

## Conclusions

This study explores the role of the solvent matrix in controlling ion yields in DAI-MS. Ion yields were measured for aerosols generated from simple binary (angiotensin II, hydrocortisone, and ammonium sulfate) and ternary (ammonium sulfate-angiotensin II) systems exposed to different RH conditioning histories. The RH dependence on ion yield is tightly coupled with the aerosol’s hygroscopic response and RH conditioning history, leading to RH-dependent and phase-dependent ion yields that are linked to aerosol liquid water content. This relationship can be quantified by relating the number of moles of solvent to the number of moles of analyte (*n*_solvent_/*n*_analyte_). Ion yield is relatively low and insensitive to droplet water content when *n*_solvent_/*n*_analyte_ is relatively low (less than ∼2) but is larger and can become highly sensitive to droplet water content at moderate and high values (*n*_solvent_/*n*_analyte_ > 2). These observations enable a critical evaluation of the factors controlling ionization by this inlet-based approach that is coherent across multiple studies and aerosol droplet sizes. A firm understanding of the ionization approach is essential for widespread use of the technique across different application domains including environmental analysis and laboratory studies of aerosol chemical composition, which often are conducted under dynamic ambient conditions. The dependencies of ion yield on RH conditioning history can be easily accounted for by normalizing the RH through condensational growth of the aerosol in a supersaturated environment. The outcomes of this study may also help to inform our understanding of inlet ionization approaches more generally and highlight some similarities with ESI.

## Supplementary Material



## Data Availability

All data underlying the figures will be made available through the University of Bristol data repository, data.bris, at https://doi.org/10.5523/bris.3subynguuhgdi2e896m4jx8sub.

## Notes

Schematic of the CK-EDB experimental approach; measured and modeled aerosol hygroscopic response; example DAI mass spectra for all studied systems; and size-dependent ion yields (PDF)

## ∇

For K. M. Risby: Department of Engineering, University of Cambridge, Trumpington Street, Cambridge, CB2 1PZ, United Kingdom
