# Supplementary material for: The Role of Aerosol Liquid Water in Droplet-Assisted Ionization Mass Spectrometry
Source: Anal Chem. 2025 Aug 31;97(36):19918–25. doi: 10.1021/acs.analchem.5c04149 (PMC12444750; doi:10.1021/acs.analchem.5c04149)
Supplement: Supplementary file 1 [file ac5c04149_si_001.pdf]

Supplementary Information

The Role of Aerosol Liquid Water in Droplet-Assisted Ionization Mass Spectrometry

Joshua Harrison, Kelvin M. Risby[a], Barnaby E. A. Miles, Thomas G. Hilditch, Jim S. Walker, and Bryan R. Bzdek*

School of Chemistry, University of Bristol, Cantock's Close, Bristol, BS8 1TS, United Kingdom

[a]now at: Department of Engineering, University of Cambridge, Trumpington Street, Cambridge, CB2 1PZ, United Kingdom

*Corresponding author: b.bzdek@bristol.ac.uk

Table of Contents

Figure S1……………………………………………………………………………………S2

Figure S2……………………………………………………………………………………S3

Figure S3……………………………………………………………………………………S4

Figure S4……………………………………………………………………………………S5

Figure S5……………………………………………………………………………………S6

Figure S6……………………………………………………………………………………S7

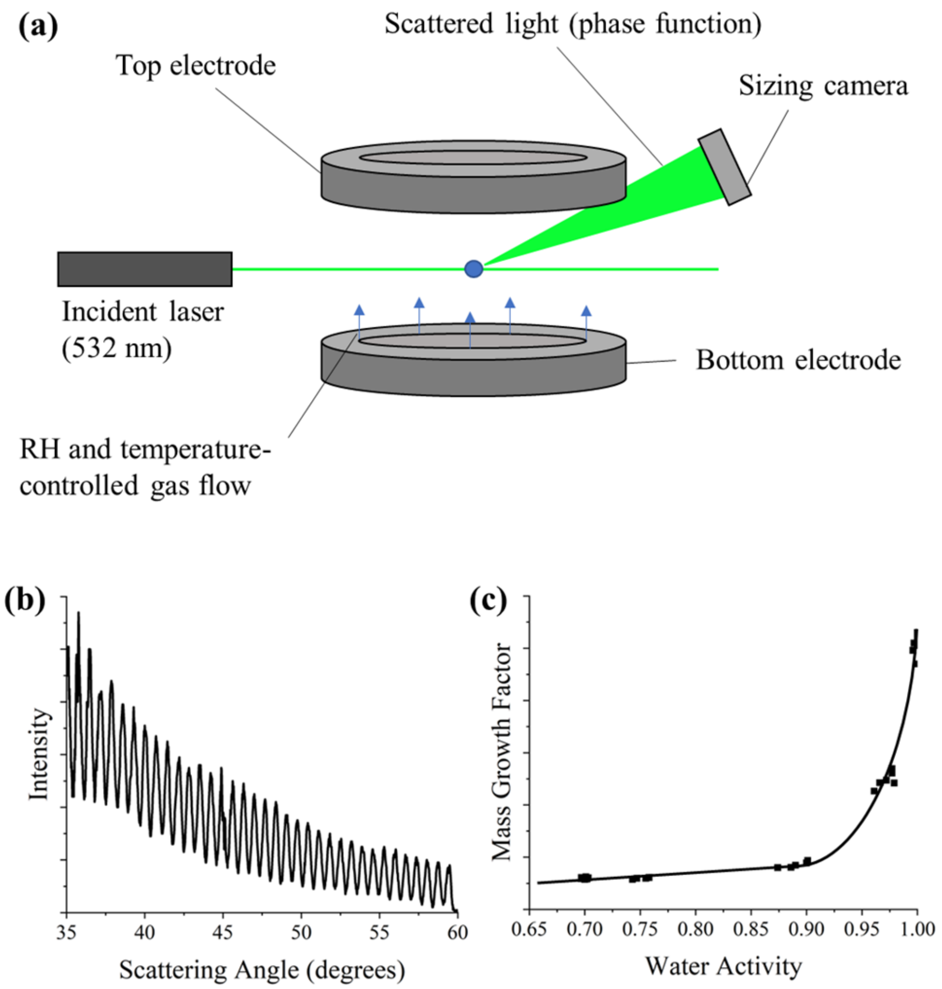

Figure S1: a) Schematic of the comparative kinetics electrodynamic balance (CK-EDB) instrument for measurement of droplet mass growth factor (MGF), b) example phase function for a levitated liquid droplet and c) plot of MGF against water activity.

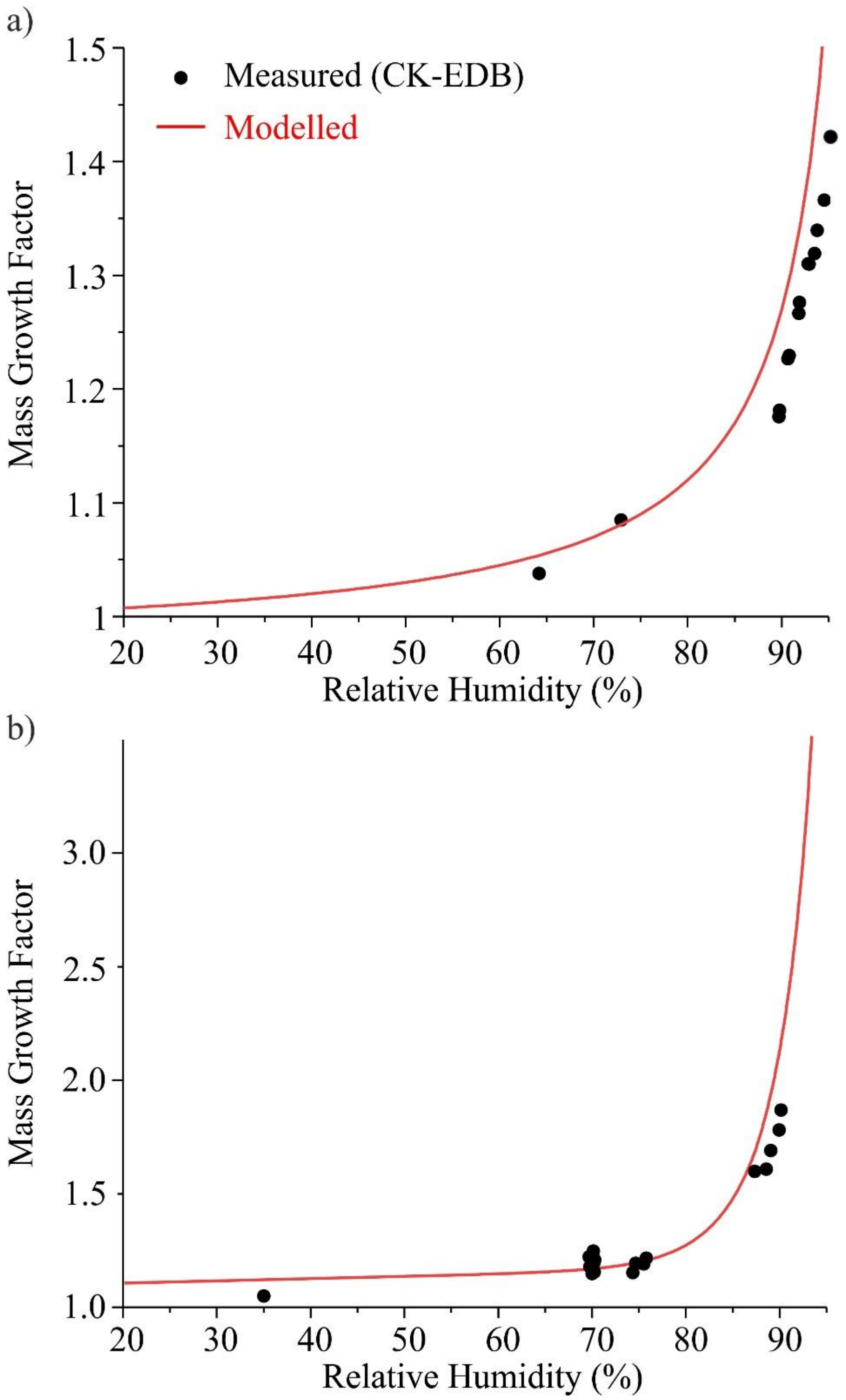

Figure S2: Measured (with the comparative kinetics electrodynamic balance) and modelled (with AIOMFAC) hygroscopicities for a) angiotensin II and b) an equimolar mixture of ammonium sulfate and angiotensin II.

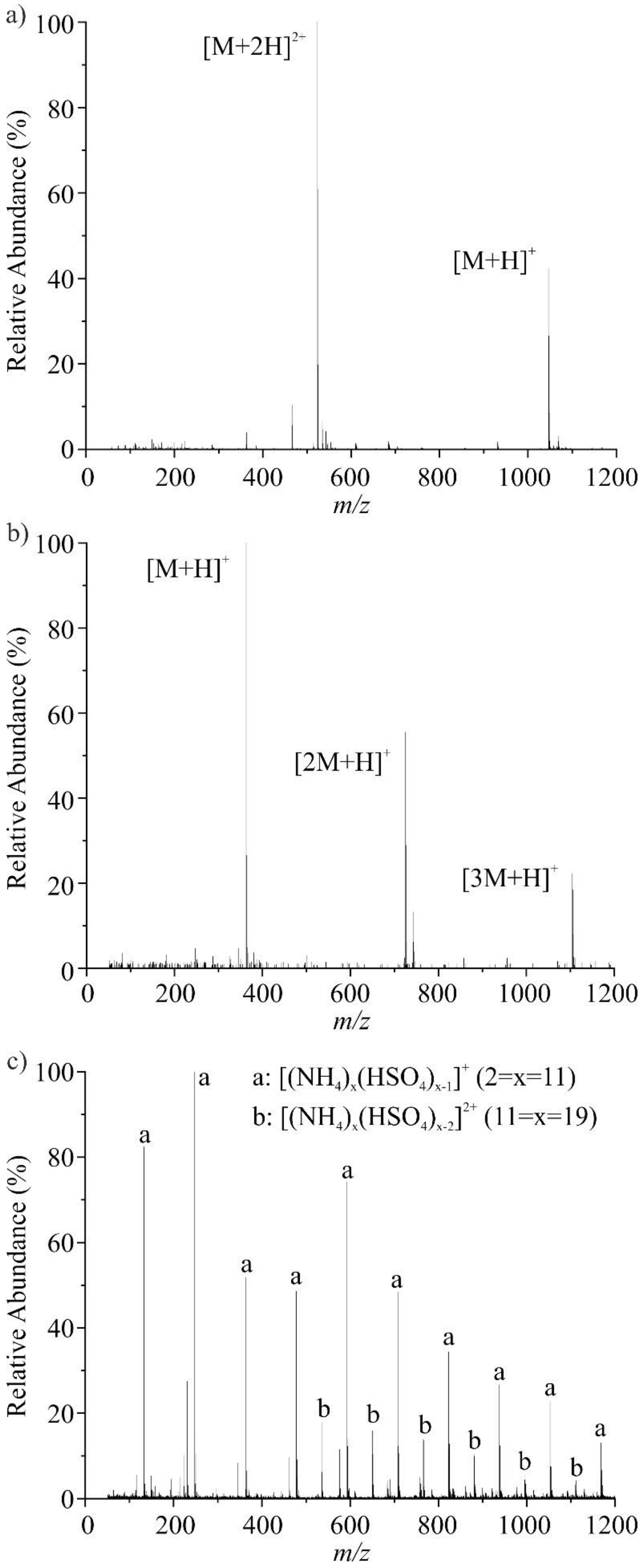

Figure S3: Example DAI mass spectra for a) angiotensin II, b) hydrocortisone, and c) ammonium sulfate. The identified peaks associated with the analyte were included in the total ion count calculation if their relative intensity was >5%.

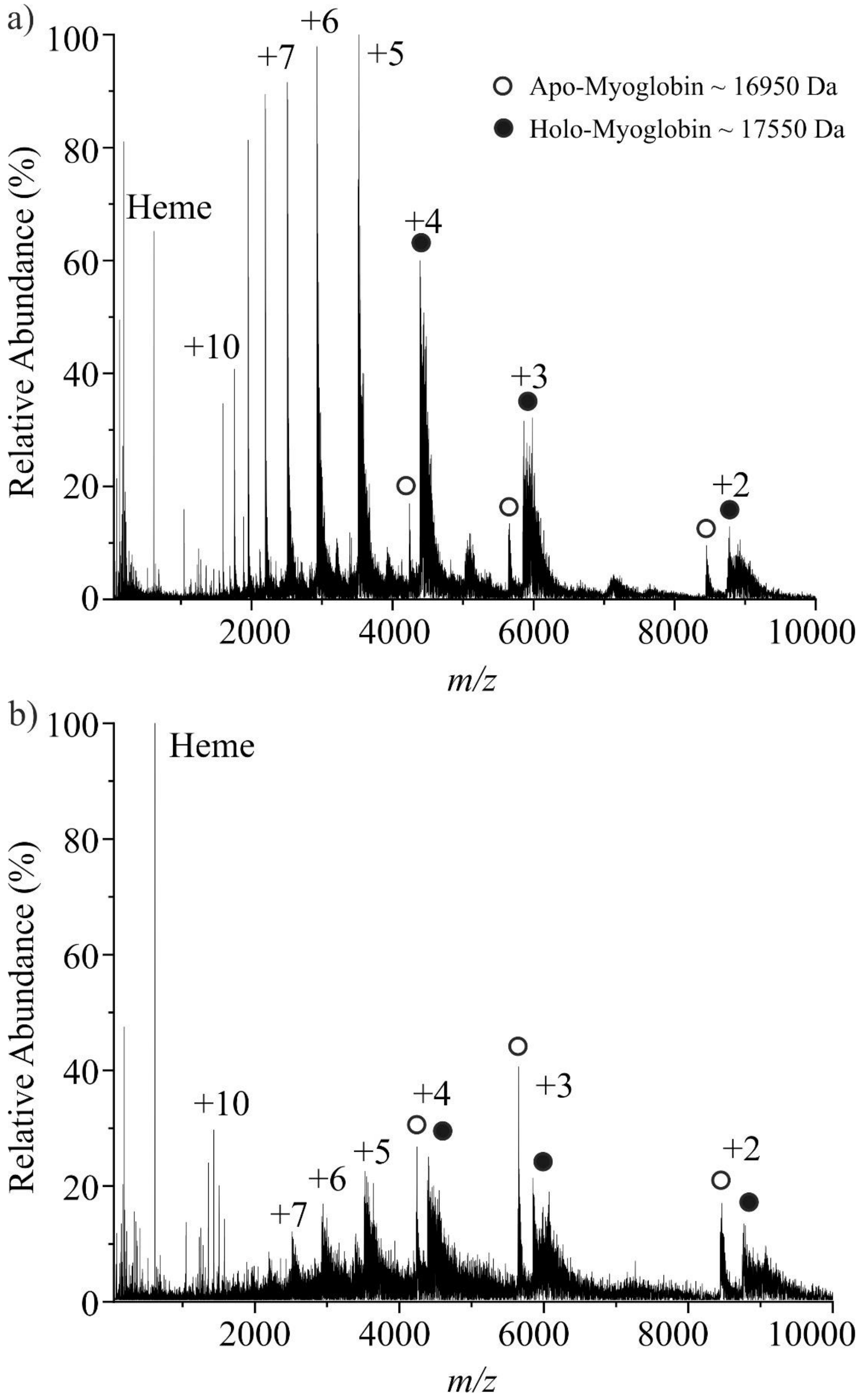

Figure S4: DAI mass spectra for equine heart myoglobin under a) wet (90% RH) and b) dry (53% RH) conditions. Higher charge states are more abundant in the mass spectrum at 90% RH. Moreover, ions associated with holo-myoglobin were more prevalent (relative to apo-myoglobin) under wet conditions. The two forms are only labelled for ions with charges +2, +3, and +4, although they are both observed in higher charge state ions.

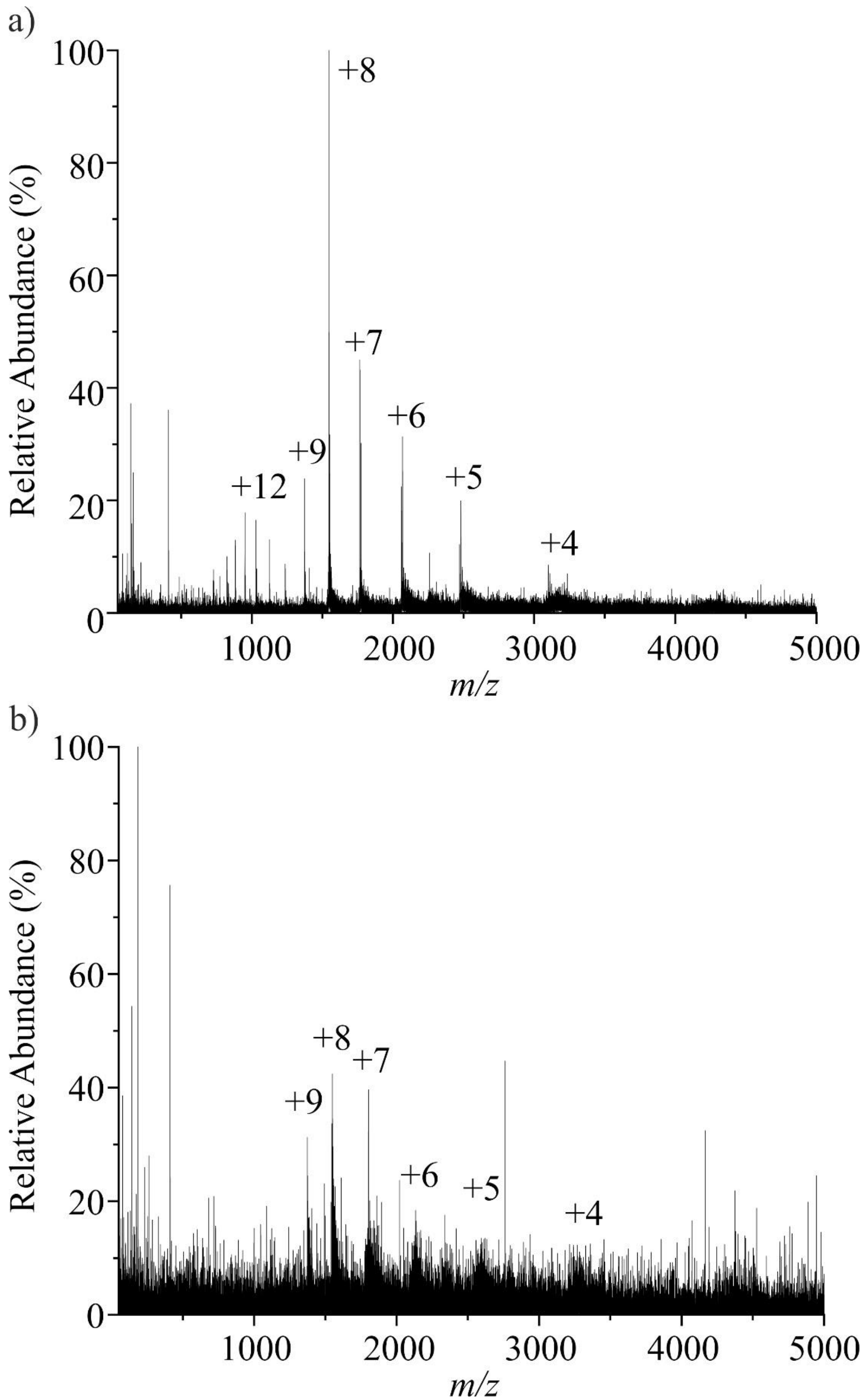

Figure S5: DAI mass spectrum for cytochrome C under a) wet (90% RH) and b) dry (33% RH) conditions. Higher charge state ions were more prevalent at 90% RH than at 33% RH.

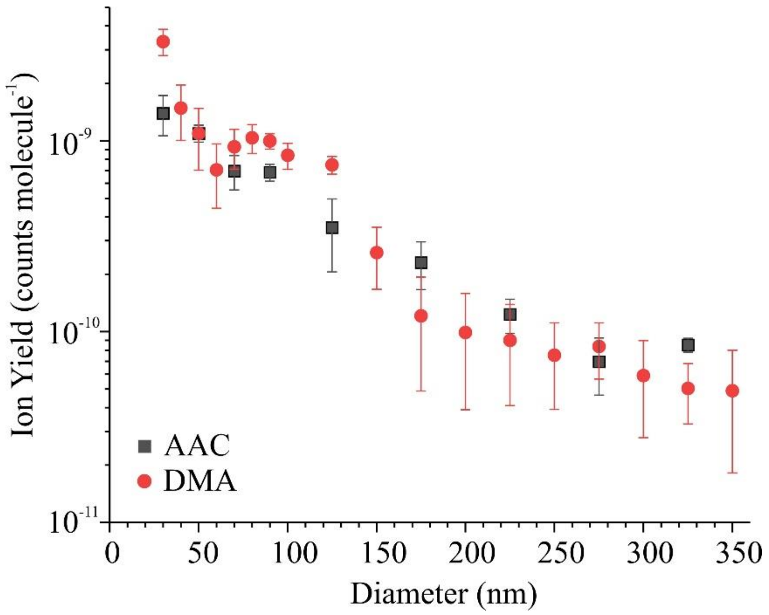

Figure S6: Ion yields for size-selected angiotensin II particles equilibrated to 70% RH before size-selection. In the experiment, particles were size-selected using an aerodynamic aerosol classifier (AAC) or a differential mobility analyzer (DMA).
